# Predictors of hospital-acquired pressure injuries in patients with complete spinal cord injury: a retrospective case–control study

**DOI:** 10.1186/s12891-023-06369-y

**Published:** 2023-04-26

**Authors:** Phumeena Balasuberamaniam, Abeer Wasim, Mopina Shrikumar, Tan Chen, Tracey Anthony, Andrea Phillips, Avery Nathens, Martin Chapman, Eric Crawford, Carolyn E. Schwartz, Joel Finkelstein

**Affiliations:** 1 Division of Orthopedic Surgery, Sunnybrook Health Sciences Centre, Toronto, ON Canada; 2 Tory Trauma Program, Sunnybrook Health Sciences Centre, Toronto, ON Canada; 3 Critical Care Medicine and Anesthesia, Sunnybrook Health Sciences Centre, Toronto, ON Canada; 4 DeltaQuest Foundation, 31 Mitchell Road, Concord, MA 01742 USA; 5 Departments of Medicine and Orthopaedic Surgery, Tufts University School of Medicine, Boston, MA USA

**Keywords:** Sacral ulcers, Vasopressors, Norepinephrine, Complete spinal cord injury

## Abstract

**Background:**

Despite current best practices, pressure injuries (PI) remain a devastating and prevalent hospital-acquired complication for patients with acute traumatic spinal cord injuries (SCIs). This study examined associations between risk factors for PI development in patients with complete SCI, such as norepinephrine dose and duration, and other demographic factors or lesion characteristics.

**Methods:**

This case–control study included adults with acute complete SCIs ASIA-A, who were admitted to a level-one trauma center between 2014–18. A retrospective review was implement using data on patient and injury characteristics, including age, gender, level of SCI (cervical vs. thoracic), Injury Severity Score (ISS), length of stay (LOS) and mortality; presence/absence of PI during their acute hospital stay; and treatment factors such as spinal surgery, mean arterial pressure (MAP) targets, and vasopressor treatment. Multivariable logistic regression evaluated associations with PI.

**Results:**

Eighty-two out of 103 eligible patients had complete data, and 30 (37%) developed PIs. Patient and injury characteristics, including age (Mean: 50.6; SD:21.3), location of SCI (48 cervical, 59%) and ISS (Mean 33.1; SD:11.8), did not differ between PI and non-PI groups. Logistic regression analysis revealed that male gender (OR:34.1; CI_95_:2.3–506.5, *p* = 0.010) and increased LOS (log-transformed; OR:20.5, CI_95_:2.8–149.9, *p* = 0.003) were associated with increased risk of PI. Having an order for a MAP > 80mmg (OR:0.05; CI_95_:0.01–0.30, *p* = 0.001) was associated with a reduced risk of PI. There were no significant associations between PI and duration of norepinephrine treatment.

**Conclusions:**

Norepinephrine treatment parameters were not associated with development of PI, suggesting that MAP targets should be a focus for future investigations for SCI management. Increasing LOS should highlight the need for high-risk PI prevention and vigilance.

## Introduction

Despite the implementation of evidence-based clinical guidelines and technological advances, the prevalence of hospital-acquired pressure injuries (PIs) remains a major healthcare concern [1]. PIs are often preventable but can be a potentially life-threatening complication caused by prolonged pressure on the skin. The Centers for Disease Control and Prevention estimate that about 2.5 million people suffer from PIs annually, and an estimated 60,000 die [2]. A recent meta-analysis found that 32% of patients with SCI experience PIs [3]. Nationally, the United States spends about $26.8 billion a year on treatment costs for PIs [2]. The development of PIs confers significant physiologic stress for patients, increasing their risk for hospital-acquired infection, prolonged hospitalization, and mortality, and moreover posing a significant cost burden on healthcare systems. This cost is expected to increase as the number of patients with complete spinal cord injuries (SCI) increases [4].

The lack of protective sensory perception and limited mobility in patients with SCIs make these patients particularly vulnerable to developing PIs [5]. In fact, Brienza et al. [6] found that the incidence of the first sacral ulcer in American Spinal Injury Association [ASIA] classification ‘A’ (complete injury) participants was about 4.5 times greater than for ASIA B or ASIA C. This makes identifying the risk factors and implementing effective prevention strategies for the development of pressure injuries in this demographic a healthcare priority [6].

The 2019 spring Trauma Quality Improvement Program Report, consisting of data acquired from a total of about 500 level-1 and 2 trauma centers in the United States and Canada, reported an average PI incidence rate of 0.7% (*N* = 294,110). The etiology of PIs is multifactorial and not solely a consequence of pressure itself. A number of risk factors have been associated with the development of PIs in critical care patients, which include, immobility, increased age, local perfusion, drugs administered, malnutrition, autonomic dysregulation and hypotension [7], cardiocirculatory management, and medical comorbidities such as diabetes mellitus [8–10]. Therefore, it would be useful to better define pertinent predisposing factors of medical origin. This can facilitate both prophylaxis and treatment of populations at risk for developing PIs, such as patients with complete SCIs. Research on risk factors for the development of PIs in patients with SCI has documented a higher PI risk with complete lesions (i.e., ASIA-A classification, pneumonia or pulmonary disease, sedative medications, low scores on the Functional Independence Measure, and longer length of stay) [11, 7, 12]. Additionally, a higher overall PI risk was associated with having had a PI during the acute rehabilitation phase [11]. Accordingly, identifying risk factors for development of PIs during acute rehabilitation would be warranted for primary prevention.

Physicians will order vasopressors following traumatic SCIs to keep the mean arterial pressure (MAP) above 85 mmHg in accordance with the American Association of Neurological Surgeons / Congress of Neurological Surgeons recommendation to increase perfusion to the spinal cord [13, 14]. However, in doing so, vasopressor administration constricts peripheral vasculature reducing blood circulation to the skin which may increase susceptibility to PI formation [8], for patients with SCI [15, 16]. The choice of vasopressor therapy and their inherent pharmacological differences could offer a causative explanation of PI development if indeed there is an association with these. This, combined with atrophy of the muscles which provide a layer of cushioning to the skin following paralysis, may increase the risk of PI [6]. Change in autonomic nervous system function in the acute phase of SCI may be especially relevant, particularly neurogenic shock, cardiac dysrhythmias, orthostatic hypotension, autonomic dysreflexia, temperature dysregulation, and hyperhidrosis [17]. Autonomic dysreflexia in SCI exerts insidious effects on cardiac function [18], and may provide an etiological link to the higher risk of heart disease in SCI [19, 20].

The primary objective of this study is to investigate the association between treatment with norepinephrine (mean rate, maximum rate, duration, and continuous versus intermittent infusions) and PI formation in patients with acute ASIA A cervical and thoracic level SCI. We only studied the role of norepinephrine as it is the primary vasopressor used specifically in the SCI population. Given that the sensorimotor recovery is largely minimal in patients with complete SCIs, we hypothesize that it might be beneficial to restrict vasopressor use to decrease the risk of PI development. A secondary objective of this research is to identify other risk factors for PI development in this patient demographic. Gaining specific insight into the factors which correlate with PI development may help develop comprehensive guidelines for PI prevention in patients with SCI.

## Methods

### Sample

This case–control study utilized a retrospective review performed on medical charts of consecutive patients with the following eligibility criteria: complete SCI ASIA-A, presenting to Sunnybrook Health Sciences Center, a Canadian level-one trauma center, over a four-year period (2014–18). We collected data on patient and injury characteristics and treatment received, including norepinephrine treatment, during their acute hospital admission. Patient and injury characteristics recorded for each patient included age, gender, location of SCI (cervical or thoracic spine), Injury Severity Score (ISS), length of stay, and mortality. Specifics of treatment received were captured and recorded for the following variables: MAP target orders, norepinephrine treatment (mean rate, maximum rate, duration, and continuous versus intermittent infusions) and whether or not the patient received surgical intervention for their acute SCI. Lastly, the presence (case) or absence (control) of sacral PIs during the patient’s acute hospital admission was recorded. All cases of sacral PI were characterized by partial or full thickness skin breakdown. The study was reviewed and approved by the Sunnybrook Health Sciences Centre Research Ethics Board [Sunnybrook SunRISE # 2152 (formerly 260–2019)]. Informed consent was waived by the Sunnybrook Health Sciences Centre Research Ethics Board [Sunnybrook SunRISE # 2152 (formerly 260–2019)] as this was a retrospective study of existing medical records.

### Statistical analysis

Descriptive statistics were used to describe the sample. Bivariate analyses assessing associations with PI included the t-test and Cohen’s d to indicate effect size (for continuous variables) and the chi-square test with Phi[2] to show effect size (for categorical variables). Potential associations with PI were then further examined using multivariable logistic regression analysis, so as to isolate each relationship while controlling for the other predictors. Three predictors were log-transformed to create more normal distributions, to minimize the influence of outliers, and to facilitate more accurate and reliable results. These predictors included hospital Length of Stay (LOS), norepinephrine mean rate, and norepinephrine total duration. Our regression models were evaluated using three versions of pseudo-R squared, plus Concordance or the C-statistic (area under the Receiver Operating Characteristic curve). The ratio of events to subjects was adequate to yield minimal bias [21, 22]. Statistical significance was defined as *p* < 0.05.

## Results

The sample included 103 people, of whom 82 were on at least one vasopressor and had complete data on all variables included in the model. Among these 82 patients included in our logistic regression, 30 (37%) had developed pressure injuries. This sample size would have sufficient power to detect a large effect-size in group differences (ß = 0.80, α = 0.05 [22]). Sixty (73%) had undergone spine surgery; 64 (78%) had been given a MAP Order > 80 mmHg; and 73 (89%) were male. The ISS was greater than 15 for 81 of 82 patients, reflecting a severe level of injury. Before log-transformation, hospital LOS had a median of 35 days; mean rate of norepinephrine Infusion had a median of 5 mcg/min; and total duration of norepinephrine Infusion had a median of 114 h (Table 1).

**Table 1:** Descriptive Statistics of Sample (*N* = 82)

	**#**	**%**		
Presence of Pressure Injury	30	37%		
Received Spine Surgery	60	73%		
Male Gender	73	89%		
Minimum Mean Arterial Pressure (MAP) Order > 80 mmHg	64	78%		
Vasopressor Treatment Received				
Epinephrine	5	6%		
Norepinephrine	82	100%		
Vasopressin	22	27%		
Phenylephrine	5	6%		
Dopamine	6	7%		
Location of Spinal Cord Injury				
Cervical	48	59%		
Thoracic	34	41%		
Comorbidity History^a^				
Diabetes	14	17%		
Peripheral Artery Disease	9	11%		
Cardiovascular Disease	18	22%		
	**Minimum**	**Maximum**	**Mean**	**Std. Deviation**
Age	16	87	50.6	21.3
Injury Severity Score (higher = greater severity)	15	75	33.1	11.8
Hospital Length of Stay in Days	1	411	52.8	63.4
Mean Rate of Norepinephrine Infusion (mcg/min)	1.3	20.9	6.0	3.8
Total Duration of Norepinephrine Infusion (hours)	2.0	4880.3	204.4	540.9

^a^Those missing a value on these conditions (2 < = N < = 3) were treated as not having a history of the condition

In the initial bivariate tests of binary predictors, only MAP Order > 80 mmHg was statistically significant. Patients with such an order had a lower incidence of PI (phi^2^ = 0.11). For the t-tests of continuous predictors, transforming the three highly skewed predictors created much more usable distributions free of notable outliers. The only significant t-test result was for LOS, log_10_ (t = 2.97, *p* = 0.004, Cohen’s *d* = 0.68) (Table 2).

**Table 2:** Results of Bivariate Relationships with Sacral Ulcer (*N* = 82)

	Chi-Square	p	ES: phi^2^	
Male	2.83	0.09	0.03	
Spine Surgery	1.12	0.29	0.01	
Minimum Mean Arterial Pressure (MAP) Order > 80 mmHg	9.00	0.00	0.11	
Comorbidity History			0.00	
Diabetes	1.46	0.23	0.02	
Peripheral Artery Disease	0.04	0.85	0.00	
Cardiovascular Disease	3.59	0.06	0.05	
	t	p	ES: Cohen's d	Mean Difference (Ulcer—No Ulcer)
Injury Severity Score (higher = greater severity)	0.24	0.81	0.06	0.65
Age	-1.38	0.17	-0.32	-6.70
Hospital LOS, log10	2.97	0.004	0.68	0.30
Norepinephrine Mean Rate, log10	1.06	0.30	0.24	0.06
Norepinephrine Total Duration, log10	1.39	0.17	0.32	0.16

*ES* Effect Size

Table 3 shows results of the logistic regression model. This model revealed that having a minimum MAP order of > 80 mmHg was associated with a reduced risk of PI, and log10 of LOS with an increased risk of PI (OR = 0.05 and 20.5, respectively). The log_10_ rate of norepinephrine treatment and log_10_ of duration remained nonsignificant. Besides meeting distributional assumptions, regression model predictors all had acceptable tolerance levels (Table 3). The model explained an estimated 33%—45% of the variance in the PI variable. Concordance, or the area under the ROC curve, was strong at 0.849.

**Table 3:** Results of Logistic Regression Analysis^a^

	**b**	**S.E**	**Wald**	**df**	**p**	**OR [Exp(b)]**	**CI**_**95**_ ** for OR**			
							**LL**	**UL**	**Tolerance**	**VIF**
Injury Severity Score (higher = greater severity)	-0.033	0.026	1.596	1	0.206	0.97	0.92	1.02	0.86	1.17
Male	3.530	1.377	6.575	1	0.010	34.11	2.30	506.48	0.91	1.10
Patient had spine surgery	0.193	0.718	0.072	1	0.788	1.21	0.30	4.96	0.94	1.06
Minimum MAP Order > 80 mmHg	-2.905	0.872	11.104	1	0.001	0.05	0.01	0.30	0.91	1.11
Hospital LOS, log10	3.020	1.015	8.849	1	0.003	20.49	2.80	149.90	0.62	1.62
Norepinephrine Mean Rate, log10	1.862	1.250	2.218	1	0.136	6.43	0.56	74.60	0.86	1.16
Norepinephrine Total Duration, log10	0.220	0.694	0.101	1	0.751	1.25	0.32	4.85	0.62	1.63
Constant	-7.239	2.738	6.987	1	0.008	0.001				

*VIF* Variance Inflation Factor

^a^Pseudo-R2 based on the Cox & Snell and Nagelkerke formulas ranged from 0.33 to 0.45; similarly, the squared correlation between model-predicted probabilities and PI was 0.36

## Discussion

The overall prevalence of PIs in patients with SCI at our institutional ICUs was 37%. This is comparable to findings on international prevalence where PIs are reported to be the most common complication of SCI, even in countries with developed healthcare systems [23]. While age and SCI itself are contributing factors to pressure ulcer prevalence, the conditions for receiving medical treatment also play a significant role [23].

Maintaining MAP targets greater than 80 mmHg was, however, seemingly protective against PI. Norepinephrine is what drives MAP, so this result suggests that there is no contraindication to driving MAP targets, in contrast to our hypothesis. Further, there was no significant association found between the treatment parameters (infusion rate and total duration) of norepinephrine and the development of PI in patients with SCI. The median rate of norepinephrine infusion in this study was 5 mcg/min (0.3 mg/hour). While a moderate-high dose (= 2.5 mg/hour) of daily norepinephrine has been associated with increased risk of PIs in other studies involving critical care patients, this association was not seen with moderate-low dose infusions (< 2.5 mg/hour) [15, 24].

Choice of vasopressor or of a combination of pressors is dependent on the individual patient’s clinical situation. Our analyses did not specifically contrast NE with combination therapy. Among the 82 patients in our model, all received NE, while 20 of these 82 patients also received vasopressin. Among all 103 patients in our data set, 86 received NE alone, none received vasopressin alone, and 24 received both. Norepinephrine vs vasopressin have inherent pharmacological differences. Norepinephrine has sympathomimetic pharmacology, being a potent stimulator of alpha receptors and also, to a lesser extent, beta receptors [25], which stimulate cardiac contractility. Vasopressin, however, acts directly on V1 receptors to produce only vasoconstriction. In septic shock, where there may be inappropriately low endogenous levels, vasopressin infusions have been shown to increase blood pressure and cardiac output with reduction in norepinephrine doses [26]. In the current study, patients were likely not exhibiting septic physiology, and the vasopressin may produce blood-pressure increase at the expense of tissue perfusion. In the large arterioles of animals, this seems to be the case [27]. Given the roles of vasopressors in the management of the patient’s overall medical condition, it would be impossible to make any definitive conclusions about one drug vs. the other. It is also probable that the use of vasopressin may only be an indicator of a worse severity of illness and thereby associated with PI development. This is a confounder in our results.

Hospital LOS was also associated with an increased risk for PI development. This is consistent with previous literature as PIs have been associated with an increased median LOS of 4.31–7.5 days [28–30]. The increased LOS may be a cause or an effect of PI development as this complication requires ongoing care in an acute setting. The severity of the SCI, completeness, and level of injury combined with additional traumatic injuries might influence the LOS. Beside the quality of the clinical management, the severity of the trauma might influence also the occurrence of complication and prolonged hospital stay. In addition, the LOS for these patients could have also been prolonged by other hospital complications and/or comorbidities that were not examined in this study, including urinary tract infections, ventilation dependence, sedation burden, pneumonia, and cardiac arrest [15]. These complications could have resulted in prolonged immobility, which is a major risk factor for developing PIs, and thus may play an unknown factor in PI development [31]. The association between increased LOS and PI development could also be reflective of a third variable, which may have not been examined in this study.

The chief difference between bivariate results and those of the multivariate model concerned male gender. Once it was considered in the presence of covariate controls, its association with PI became stronger. This finding is consistent with previous studies as male patients are often reported to have a higher incidence of PIs compared to female patients [32, 33]. This gender difference for PI development could be reflective of various factors including differences in age, BMI, and hormonal differences such as increased estrogen, have been associated with decreased incidence of PIs and increased wound healing [34]. Because almost all of the patients had a severe injury, adjusting for their ISS had limited value in accounting for this severity. It may be that males are more likely to have severe SCI or compound medical problems that result in longer LOS. Such factors, if controlled, might well reduce the observed gender difference in PI risk. Future research might over-sample females in a study of risk factors for PI in SCI to have more balanced groups in evaluating the effect of gender.

### Clinical implications

In accordance with the clinical guidelines for SCI, clinicians seek to increase perfusion to the spinal cord. Our study findings show no detrimental effect of MAP orders on PI development. Thus, MAP orders were protective against PI, contrary to our hypothesis.

Pittman et al. [35] investigated avoidable versus unavoidable factors for hospital-acquired PIs in critical care patients. Unavoidable PIs were defined as those that developed in spite of consistent documentation of evidence-based preventive interventions [35]. They found that for each 1-day increase in stay, the odds of developing an unavoidable pressure ulcer increased by 4% [35]. Consistent with previous literature, our study found that patients with hospital-acquired PIs typically had a longer stay [36]. Therefore, it is worth revisiting best-practice skin care and mattress protocols for patients with SCI. Preventing secondary complications, including PIs, falls under every health discipline’s domain, but a large portion of that lies within nursing’s realm. The following strategies have been implemented throughout the Trauma, Spine and Neurosurgical programs at our institution [37–39]:

- Development of an Acute Spinal Cord Injury Critical Care Admission Order Set with SCI best practice guidelines at each patient's bedside. The order set encompasses the expected blood pressure targets and frequency of spinal cord testing, as well as orders referring to skin care, frequency of turning, use of heel lift boots, and choice of mattress, to ensure that all aspects of care for the newly spinal cord injured are addressed as early as possible.
- Each patient with SCI on admission has a laminated Turn Clock at their bedside as a visual reminder that turning every two hours is required as well as a checklist of nursing guidelines to prevent and monitor signs of a pressure ulcer. Modern management includes wearables that measure the individual’s sensitivity, and adapts their position accordingly.
- New policy stating transfer boards are not to be left on during scans or x-rays.
- Increased use of wedges for positioning patients instead of pillows.

### Limitations and future research

As this study looked at data retrospectively, there are some limitations worth noting. Firstly, indications for the use of norepinephrine could have stemmed from reasons other than SC perfusion. Norepinephrine is commonly administered to patients in the ICU to avoid hypotension associated with myocardial injury, kidney injury and death [40]. The selection of norepinephrine and duration must also consider the increased cardiogenic complications in the elderly [41]. Second, the present study was not able to address whether epinephrine application is related to PI occurrence due to the small sample size of patients who received epinephrine. Future research might focus on this relationship. Additionally, several relevant factors were not accounted for; these include duration of time on a hard spine board, use of preventative dressings, and number of patient-turns. However, these factors are expected to be quite consistent across patients, given institutional guidelines. Without significant variability, they would likely do little to explain the PI outcome. Further, ventilation days and sedation burden were not data points in this study and may be unknown factors in PI development. Finally, due to the relatively small sample size, retrospective design, and adjustment for only a subset of possible risk factors, the effect estimates should be interpreted with caution, since the OR can then reach very high or small values with huge CIs.

Since PI complications are one of the most common complications among this population [42], economic research focused on PI-associated resource utilization and costs of care in patients with SCI is pertinent. Currently, the majority of current cost analyses focus on rehabilitation, with overall mean first-year costs reported to be the greatest at $222 thousand USD per patient [43]. Direct annual medical costs associated with treating PIs in veterans with SCI in the United States alone have been estimated at $89 million USD and between $173 million and $316 million CAD in community-dwelling SCI individuals in Canada [42]. A detailed cost-analysis of PI prevention and care in patients with SCI in acute care settings may help establish the urgency required for institutional reform. Lastly, while this retrospective review enables us to identify associations, such as gender or hospital LOS, it does not specify causation. This may warrant future prospective randomized controlled trials.

## Conclusions

In summary, the average dose and duration of norepinephrine used in patients with SCIs were not associated with an increased risk of developing PIs. Factors associated with an increased risk for PI development included MAP < 80 mg, increased hospital LOS and male gender. Thus, adherence to preventive guidelines and vigilant monitoring and treatment of PIs in males with complete SCI should be reinforced within institutions. MAP targets should be a focus for future investigations for SCI management. Pressure/sacral ulcer care is undoubtedly complex and costly. Promoting adherence to preventive interventions requires a systematic approach that involves assessing an organization's readiness for change. Readiness requires both the capability and motivation to make the change. Therefore, promoting multidisciplinary understanding about hospital-acquired PIs and continually reviewing and adapting best practice guidelines is essential to reduce the prevalence of hospital-acquired PI in patients with SCI.

## Data Availability

The study data are confidential and thus not able to be shared. Interested readers should contact the corresponding author of this study.
